# Falls and Physical Activity in Persons with Multiple Sclerosis

**DOI:** 10.1155/2012/315620

**Published:** 2012-08-23

**Authors:** J. J. Sosnoff, B. M. Sandroff, J. H. Pula, S. M. Morrison, R. W. Motl

**Affiliations:** ^1^Department of Kinesiology and Community Health, University of Illinois at Urbana-Champaign, 301 Freer Hall, 906 South Goodwin Avenue, Urbana, IL 61801, USA; ^2^Illinois Neurologic Institute, University of Illinois College of Medicine at Peoria, Peoria, IL 61605, USA; ^3^Department of Physical Therapy, Old Dominion University, Norfolk, VA 23529, USA

## Abstract

*Objectives*. To examine the association between fall history and physical activity using an objective measure of physical activity (i.e., accelerometry) in persons with multiple sclerosis. *Design*. A community-based sample of 75 ambulatory persons with multiple sclerosis volunteered for the investigation. Participants self-reported fall history in the last year, underwent a neurological exam to determine Expanded Disability Status Scale (EDSS) score, and wore an accelerometer around the waist for 7 consecutive days to determine physical activity. *Results*. Overall, 37 persons (49.3% of the sample) reported falling in the last year with 28 of the 37 falling more than once. Persons who fell in the last year had a significantly lower number of steps/day than nonfallers (3510 versus 4940 steps/day; *P* < .05). However, when controlling for disability status there was no statistically significant difference between fallers and nonfallers (4092 versus 4373 steps/day; *P* > .05). *Conclusions*. Collectively, the findings suggest that fall history may have little impact on current physical activity levels in persons with multiple sclerosis.

## 1. Introduction

Multiple sclerosis (MS) is a chronic and disabling neurologic disease that is common among adults worldwide and in the United States. The relapsing-remitting clinical disease course, accounting for nearly 85% of cases, involves episodes of focal inflammation in the central nervous system (CNS) [1] that often result in demyelination and transection of axons. This progressive axonal damage produces conduction delay along neuronal pathways throughout the CNS [2] and eventually results in heterogeneous symptoms including visual impairment, vertigo, impaired proprioception, decreased vibration sense, muscle weakness, and spasticity [3].

Given those symptoms, it is not surprising falls are common in persons with MS [4–7]. Injuries resulting from falls in persons with MS routinely require medical attention [6, 8, 9], and falls have been associated with activity curtailment and subsequent deconditioning [10, 11]. One study reported that 63.5% of persons with multiple sclerosis have a fear of falling (FoF) and, of those individuals, 82.6% reported activity curtailment [10]. Another recent study documented that approximately 75% of community-dwelling persons with MS who have fallen in the last 6 months self-reported activity restriction due to concerns about falling [11]. 

One limitation of these previous investigations is that the quantification of reduced physical activity behavior was based exclusively on self-report surveys with unknown psychometric properties. Such survey metrics of activity might reflect self-report response biases and inaccuracy associated with memory and recall. To that end, accelerometry represents a valid and objective approach for measuring physical activity behavior as steps/day in persons with MS [12, 13]. Indeed, some researchers have noted that accelerometers have the capacity for providing a gold standard measure of free-living activity in this population [12, 13]. Accordingly, this investigation examined the association between fall history and physical activity, employing objective measures (i.e., accelerometry). We hypothesized that persons with multiple sclerosis who previously experienced a fall would demonstrate lower physical activity levels as indexed by steps/day compared to nonfallers.

## 2. Methods

### 2.1. Participants

The participants included 75 community dwelling persons with a neurologist-confirmed diagnosis of MS based on either the Posner or revised McDonald criteria [14]. Inclusion criteria involved having the ability to walk independently or with a cane, crutch, or walker; comprehend written and spoken English; relapse-free for 30 days. Participants were divided into two groups based on previous fall history (fallers and nonfallers), and a fall was defined as an event where the participant unintentionally came to rest on the ground or a lower level [4]. 38 participants (51%) did not experience a fall over the past 12 months and 37 participants (49%) reported experiencing at least one fall within the same period. 

### 2.2. Procedures

This study was approved by a University Institutional Review Board and, upon arrival to the neurology clinic, all participants provided written informed consent. Participants further provided demographic information including age, gender, duration of MS, and incidence and number of falls in the past 12 months. All participants then underwent a clinical exam by a neurologist who then generated an expanded disability status scale (EDSS; [15]) score. In brief, the EDSS score reflects function in eight functional systems including the pyramidal, cerebellar, brain stem, sensory, bowel and bladder, visual, mental, and other. Those system scores plus ambulatory performance are combined into an EDSS score that ranges from 0–10 with 0 representing no neurological impairment, 4.0 reflecting the onset of walking impairment, 6.5 corresponding to the requirement of bilateral assistance (e.g., crutches or walker), and 10.0 is death due to MS [15, 16]. Participants were instructed to wear the accelerometer (Health One Technology, Actigraph Model GT3X) on an elastic belt around the waist, with the unit situated on their non-dominant hip, during the waking hours over a 7-day period. The 7-day period was chosen to capture a reliable index of usual physical activity in persons with MS [17]. This wear period was verified by visual inspection of the data and its correspondence with a log that participants maintained for recording the time the unit was worn. Activity was quantified as the number of steps/day. Steps/day were determined by Actigraph software (Health One Technology, Fort Walton Beach, FL). In essence, when the vertical acceleration exceeds a certain threshold, a step is recorded. 

### 2.3. Statistical Analysis

All analyses were completed using SPSS version 19.0 (SPSS Inc, Chicago, IL) and significance was set at *P* < 0.05. Values are reported as mean (standard deviation) unless otherwise noted. Differences in continuous demographic variables were examined utilizing independent-samples *t*-tests. Differences in noncontinuous variables were examined with Mann-Whitney test. To determine whether fall history influenced physical activity, several analyses were performed. First, a one-way ANOVA with group (i.e., fallers versus nonfallers) as the between-subject factor was conducted. Second, given that EDSS score was greater in the fall group, an ANCOVA with fall group as the between-subjects factor and EDSS as the covariate was conducted. We further expressed between-group differences in mean scores using Cohen's *d,* and values of .2, .5, and .8 were used for qualifying small, moderate, and large magnitude effect sizes, respectively [18]. Lastly, Spearman rho correlations were conducted to characterize the associations between EDSS, number of falls, and physical activity. There was no missing data within the investigation. 

## 3. Results

Demographic variables as a function of group are reported in Table 1. In brief, the average age of the sample (*n* = 75) was 52.1 (SD 12.1) years and the sample had MS for an average of 12.8 (SD 10.2) years. In terms of disability status, EDSS scores ranged from 2.0 to 6.5 with a median of 4.0 and Inter-quartile range of 3.0. 84% of the sample had relapse remitting course of MS. The fall group had a greater median disability (EDSS = 6.0) compared to the nonfall group (EDSS = 3.5) (*U*(75) = 429; *z* = −2.9; *P* < .05). There were no differences in age, MS duration, MS type, or employment status between groups (*P*'s > .05) (see Table 1). 

In the overall sample, level of activity ranged from 419 to 13,136 steps per day with an average of 4,234 (SD 2567). The fall group took significantly fewer steps/day than the nonfall group (3510 (SD 2023) versus 4940 (SD 2856), respectively; [*F*(1,73) = 6.2; *P* < .05; *d* = 0.58] (See Table 1). There were no group differences in steps/day when controlling for disability status (fall group (4092 steps/day SE 325.5) versus nonfall group (4373.5 steps/day SE 320.9); [*F*(1,72) = 0.4; *P* > .05; *d* = 0.11]. 

The correlation analysis revealed that there was a significant correlation between EDSS and steps/day (*ρ* = −0.73; *P* < .05) and between EDSS and actual number of falls in past 12 months (*ρ* = 0.31; *P* < .05). There was not a significant correlation between steps/day and number of falls in past 12 months (*ρ* = −0.15; *P* > 0.05). 

## 4. Discussion

The current investigation examined the association between fall history and an objective measure of physical activity (e.g., acclerometry) in persons with multiple sclerosis. Congruent with the primary hypothesis, persons with multiple sclerosis who experienced a fall in the previous year took significantly fewer steps/day compared to nonfallers with the difference being moderate in magnitude based on effect size. However, when controlling for disability status, previous fall history had no impact on physical activity levels. This observation is in contrast to the notion that persons with MS self-regulate (i.e., reduce) physical activity after experiencing a fall [10, 11]. While this result demonstrating a lack of physical activity differences between fallers and nonfallers would appear to be in contrast to previous observations [10, 11], there is evidence of a disassociation between fall status and ambulatory activity in older adults [19]. Other researchers [20] too have reported no differences in walking activity between older adults who had fallen during a hospitalization and those who had not fallen. As such, the current observations indicate that the association between falls and physical activity in persons with MS might not be as straightforward as previously postulated. 

There are numerous potential reasons why there was no difference in physical activity between fallers and nonfallers after controlling for disability status. One such reason involves previous investigations providing a single self-report item as the sole indication of physical activity in persons with multiple sclerosis [10, 11]. Participants who responded affirmatively to the question “are there things you do not do because of concerns of falling?” were considered to have undergone reductions of physical activity behavior [10, 11]. In contrast, reports that have found no association between fall status and activity levels have utilized objective quantification methods (e.g., accelerometry) of activity [20]. Consequently, it is possible that there is a disassociation between self-report measures of activity curtailment and objective measures of physical activity within this population. One mechanism that provides a potential explanation includes the possibility that participants changed the type of physical activity they engaged in to minimize their risk of falls, but did not reduce their overall level of physical activity. For instance, a participant might refrain from going for walks outside in bad weather (e.g., ice, rain, etc.) due to concerns of falling and subsequently replace this fall-prone activity with walking indoors (i.e., a behavior less prone to falls). This hypothetical behavior might lead to an affirmative self-report response of activity curtailment, but minimal change in physical activity objectively measured by an accelerometer. 

Another possibility why the current observation does not align with previous reports is that within the current investigation the time period between a fall and the assessment of physical activity was too great to detect reductions in physical activity. Indeed, within the current investigation physical activity was not quantified directly following a fall, but rather at some unknown time up to 12 months after a fall. Although this is a valid criticism, it should be highlighted that other researchers [20] reported no difference in walking activity immediately following a fall between older adults who had fallen while at the hospital and those that had not fallen. 

Lastly, the discrepant findings could result due to differences in demographics from previous reports. However, the current sample is comparable with those in previous reports. For instance, the fall incidence reported here (~50%) is similar to previous investigations [4–6]. Additionally, the amount of physical activity observed here is similar to previous reports [17, 21]. The similarity between the current investigation and previous reports in fall incidence and physical activity suggests that, although the current sample (*n* = 75) is smaller than previous reports, it is similar to the general MS population.

Regardless of the reason for the discrepant results, it is important to understand whether there is a reduction in physical activity due to falls or concerns about falls in persons with multiple sclerosis, since a reduction in physical activity might be indicative of a disuse-disability cycle [10]. The disuse-disability cycle suggests that reduced physical activity leads to further physiological deconditioning and subsequent disability progression and dependence [22], and there is preliminary evidence of this in persons with multiple sclerosis [23]. Further work is necessary to examine whether this reduction of physical activity with disability progression is indeed influenced by falls. 

The strong association between disability and physical activity observed here is congruent with previous reports [24–26], and the bivariate correlation analysis indicated that accelerometer output was better related with disability than fall status. There have been numerous explanations for this association. One possibility is that multiple sclerosis symptoms (e.g., fatigue, spasticity, muscle weakness, etc.) that are often more severe in persons with greater disability, make physical activity less likely [27]. It has been suggested that walking and balance impairment common in MS make physical activity difficult [26].

A limitation of the current investigation is that physical activity was only quantified as average steps/week. As a result, any physical activity that was not walking (i.e., swimming, bicycling, or gardening) was not captured. However, it should be noted that walking is the most common type of self-selected physical activity by persons with multiple sclerosis [28]. An additional limitation is the cross-sectional nature of the investigation. A longitudinal design would be more appropriate to determine the association between falls and physical activity in persons with MS. There is great promise in this approach given that accelerometery as a measure of physical activity has been successfully implemented in persons with MS in longitudinal investigations [29]. Lastly, participants were asked to report the number of falls in the last 12 months. It is possible that this time period led to an under reporting of falls. The appropriate timeframe to report falls in persons with multiple sclerosis or other populations is not clear [11, 30] and recall measures are suspect in populations with documented cognitive impairment. However, there is data to suggest that fall recall in persons with multiple sclerosis is relatively accurate[5], and the similarity between the fall incidence in the current investigation of 49.3% and previous reports [5, 6, 31] makes this less likely. 

Although it is known that falling is a significant issue among persons with multiple sclerosis [4, 6, 8], this is the first investigation to objectively measure physical activity as a function of fall history in this population. The current findings are in contrast to previous investigations which relied on self-report measures to quantify reductions in physical activity behavior. Future work should further explore the relationship between falling and physical activity in persons with multiple sclerosis. 

## Figures and Tables

**Table 1:** Participant demographics and physical activity as a function of group.

Variable	Nonfallers (*n* = 38)	Fallers (*n* = 37)	*P*	*d*
Age (years)	50.1 (13.9)	53.4 (10.0)	0.35	−0.27
MS Duration (years)	11.0 (9.6)	14.6 (10.6)	0.13	−0.36
MS Type	35 RR; 3 P	31 RR; 6 P	0.97	—
Gender (female/male)	31/7	28/9	0.54	—
EDSS (median, IQR)	3.0 (2.0)	5.0 (4.0)	0.003	−0.81
Employment (% fully/partial/unemployed)	62.2/8.3/29.7	47.4/13.2/39.5	0.24	—
Steps/day	4940 (2856)	3510 (2023)	0.02	0.58

Note: All data presented as mean (SD); EDSS: expanded disability status scale; IQR: Inter-quartile range; RR: Relapse remitting MS; P: Progressive.
